# βklotho is essential for the anti‐endothelial mesenchymal transition effects of *N*‐acetyl‐seryl‐aspartyl‐lysyl‐proline

**DOI:** 10.1002/2211-5463.12638

**Published:** 2019-04-22

**Authors:** Rongfen Gao, Keizo Kanasaki, Jinpeng Li, Munehiro Kitada, Toshiro Okazaki, Daisuke Koya

**Affiliations:** ^1^ Department of Diabetology & Endocrinology Kanazawa Medical University Uchinada Japan; ^2^ Department of Hematology & Immunology Kanazawa Medical University Uchinada Japan; ^3^ Division of Anticipatory Molecular Food Science and Technology Medical Research Institute Kanazawa Medical University Uchinada Japan

**Keywords:** AcSDKP, EndMT, FGFR1, fibrosis, KLB

## Abstract

Endothelial–mesenchymal transition (EndMT) has emerged as an essential bioprocess responsible for the development of organ fibrosis. We have previously reported that fibroblast growth factor receptor 1 (FGFR1) is involved in the anti‐EndMT effect of *N*‐acetyl‐seryl‐aspartyl‐lysyl‐proline (AcSDKP). FGFR1 is expressed on the cell membrane and performs its biological function through interaction with co‐receptors, including βklotho (KLB). However, it remains unknown whether KLB is involved in the anti‐EndMT effects of AcSDKP. Here, we demonstrated that AcSDKP increased KLB expression in an FGFR1‐dependent manner and that KLB deficiency induced AcSDKP‐resistant EndMT via the induction of the mitogen‐activated protein kinase (MAPK) pathway. In cultured endothelial cells, AcSDKP increased KLB protein level in an FGFR1‐dependent manner through induction of the FGFR1–KLB complex. KLB suppression by small interfering RNA transfection did not affect FGFR1 levels and resulted in the induction of EndMT. In contrast to the EndMT observed under FGFR1 deficiency, the EndMT induced by KLB suppression was not accompanied by the induction of Smad3 phosphorylation; instead, KLB‐deficient cells exhibited induced activation of the MAPK/extracellular signal‐regulated kinase (ERK) kinase (MEK) and ERK pathways. Treatment with the specific MEK inhibitor U0126 diminished KLB deficiency‐induced EndMT. Consistent with this finding, AcSDKP did not suppress either EndMT or MEK/ERK activation induced by KLB deficiency. Application of either FGF19 or FGF21 synergistically augmented the anti‐EndMT effects of AcSDKP. Taken together, these results indicate that endogenous peptide AcSDKP exerts its activity through induction of the FGFR1–KLB complex in vascular endothelial cells.

**Abbreviations**
AcSDKP: *N*‐acetyl‐seryl‐aspartyl‐lysyl‐proline
EndMT: endothelial–mesenchymal transition
ERK: extracellular signal‐regulated kinase
FGF: fibroblast growth factor
FGFR1: fibroblast growth factor receptor 1
FSP1: fibroblast‐specific protein 1
HMVECs: normal human dermal microvascular endothelial cells
KLA: αklotho
KLB: βklotho
MAPK: mitogen‐activated protein kinase
MEK: extracellular signal‐regulated kinase kinase
PLA: proximity ligation assay
SM22α: smooth muscle protein 22α
TGFβ: transforming growth factor β
αSMA: smooth muscle α‐actin

Endothelial–mesenchymal transition (EndMT) was initially described in the embryonic development of the heart 1. EndMT is characterized by the loss of endothelial cell markers (e.g., VE‐cadherin and CD31) and the acquisition of mesenchymal/fibroblast features such as the expression of smooth muscle α‐actin (αSMA), smooth muscle protein 22α (SM22α), fibroblast‐specific protein 1 (FSP1), and vimentin. Transforming growth factor βs (TGFβs) are considered to be the pivotal cytokines driving EndMT through the activation of the Smad signaling pathway 2, 3. It has been well documented that fibroblast growth factor receptor 1 (FGFR1) acts as the key inhibitor of EndMT by inhibiting TGFβ/Smad signaling 4, 5, 6. EndMT has emerged as a crucial source of extracellular matrix‐producing mesenchymal cells that are responsible for the development of fibrosis in involved organs, including the kidney and heart 7, 8. Therefore, the identification of approaches targeting EndMT is of great clinical potential for the development of novel therapeutic agents.

*N*‐acetyl‐seryl‐aspartyl‐lysyl‐proline (AcSDKP), an endogenous tetrapeptide, has been shown to be a substance that displays anti‐EndMT and antifibrotic effects both *in vitro* and *in vivo* by our group and other groups 4, 9, 10. AcSDKP is produced from the N‐terminal peptide sequence of thymosin β4 by the cleavage activity of prolyl oligopeptidase. AcSDKP is normally present in plasma and is exclusively hydrolyzed by angiotensin‐converting enzyme. We reported that AcSDKP inhibited TGFβ/Smad signaling 11 and displayed antifibrotic effects in various fibroproliferative rodent models in experimental intervention studies 4, 9, 10, 12. The restoration of FGFR1 expression induced by AcSDKP was later identified as the essential event responsible for the inhibition of TGFβ/Smad signaling as well as for the subsequent EndMT 4, 9. However, the molecular regulation of the anti‐EndMT effects of AcSDKP through FGFR1 remains unclear due to the complexity of FGFR1 biology.

The klotho family is a family of newly defined proteins that is composed of αklotho (KLA) 13, βklotho (KLB) 14, and γklotho 15. Klotho proteins are type I single‐pass transmembrane proteins and share homology to family 1 β‐glycosidases. The intracellular part of klotho proteins is very short, while the extracellular domain is relatively long and is composed of two internal repeats, termed KL1 and KL2 16. The main roles of membrane‐bound klotho proteins rely on their functions as co‐receptors of FGFRs, facilitating the binding of FGF19 subfamily ligands to FGFRs (e.g., KLA for FGF23 and KLB for FGF19/FGF21) 17, 18. The FGF19 and FGF21 have been emerged as essential metabolism‐regulating factors during the past decades. Tomlinson *et al*. 19 reported that the FGF19 transgenic mice were resistant to adiposity and displayed increased metabolic rate. Furthermore, FGF21 has been implicated in the stimulation of glucose uptake as well as in the resistance to diet‐induced obesity 20. The subsequent *in vitro* studies demonstrated that the KLB acts as a co‐receptor for FGF19 and FGF21, both of which exhibited low affinity to their corresponding FGFRs 21, 22, 23. In addition, Adams and colleagues provided clear evidence regarding the involvement of KLB in regulating the metabolic processes of FGF19/21 in experimental animals 24, 25.

Among the three of klotho family proteins, KLA has attracted considerable attention in studies due to its anti‐aging property 13. The functions of KLA in endothelial cells as well as cardiovascular disorders have been widely studied 26, 27, 28, 29. In contrast, the biological role of KLB is not well analyzed. The expression of KLB was detected in human umbilical vein endothelial cells 30 and human brain microvascular endothelial cells 31 and contributes to blood–brain barrier formation. However, the contribution of KLB to EndMT and fibrotic disorders remains poorly understood. Therefore, the roles of KLB in EndMT as well as its responses to AcSDKP treatment were investigated in the current study.

## Materials and methods

### Reagents and antibodies

The mouse monoclonal anti‐FGFR1 (ab823), mouse monoclonal anti‐vimentin (ab8978), and rabbit polyclonal anti‐αSMA (ab5694) antibodies were purchased from Abcam (Cambridge, UK). The human neutralizing anti‐FGFR1 (MAB765) and goat polyclonal anti‐human‐KLB (AF5889) antibodies were obtained from R&D Systems (Minneapolis, MN, USA). The rabbit polyclonal anti‐phospho‐Smad3 (s423 and s425; 600‐401‐919) antibody was bought from Rockland Immunochemicals (Gilbertsville, PA, USA). The mouse monoclonal anti‐β‐Actin (A2228) antibody was from Sigma (St. Louis, MO, USA). The rabbit polyclonal anti‐SM22α (NBP1‐33003) and rabbit monoclonal anti‐VE‐cadherin (NBP1‐43347) antibodies were obtained from Novus Biologicals (Littleton, CO, USA). The rabbit polyclonal anti‐S100A4 (also known as FSP1; PRB‐497P) antibody was from Santa Cruz Biotechnology (Dallas, TX, USA). The following antibodies and reagents were from Cell Signaling Technology (Danvers, MA, USA): the rabbit anti‐phospho‐FGFR1 (#3471) antibody, the rabbit polyclonal anti‐p44/42 mitogen‐activated protein kinase (MAPK; extracellular signal‐regulated kinase [ERK] 1/2) antibody (#9102), the rabbit polyclonal anti‐phospho‐ERK1/2 (Thr202/Tyr204) antibody (#9101), the rabbit polyclonal anti‐phospho‐MAPK/ERK kinase (MEK)1/2 (Ser217/221) antibody(#9121), the rabbit polyclonal anti‐MEK1/2 antibody (#9122), and the MEK inhibitor U0126 (#9903). Human TGFβ2 was purchased from PeproTech (Rocky Hill, NJ, USA).

### Cell culture and treatment

Normal human dermal microvascular endothelial cells (HMVECs, CC‐2516; Lonza, Basel, Switzerland) were maintained in EBM‐2 medium supplemented with EGM‐2 (5.5 mmol·L^−1^ glucose, FBS, hFGF‐b, VEGF, R‐IGF‐1, hydrocortisone, ascorbic acid, hEGF, GA‐1000, and heparin; Lonza, Alpharetta, GA, USA) as described previously 4. When the cells reached 70–80% confluence, they were subjected to the treatment with neutralizing FGFR1 antibody (N‐FGFR1; 1.5 mg·mL^−1^) or TGFβ2 (5 ng·mL^−1^) as indicated, with or without preincubation for 2 h with AcSDKP (100 nm) and/or either FGF19 or FGF21 treatment (100 ng·mL^−1^).

### Transfection experiments

As previously described 4, 32, subconfluent HMVECs cultured in serum‐free medium (a mixture of Humedia‐EB2 [KE‐23505] in serum‐free RPMI 1640 medium [Nacalai Tesque, Kyoto, Japan], 1 : 3 ratio) were transiently transfected with KLB small interfering RNA (siRNA; 100 nm; Invitrogen, Carlsbad, CA, USA) using Lipofectamine 2000 (Invitrogen) according to the manufacturer's instructions. Six hours later, the medium was replaced with an experimental medium (a mixture of HuMedia‐MVG [KE‐6550] in serum‐free RPMI 1640 medium at a 1 : 3 ratio). At 48 h, after washing with PBS, the cells were harvested for further use.

### Multiplex staining

Multiplex staining was performed by the opal *in situ* kit (PerkinElmer, Waltham, MA, USA) according to the manufacturer's instructions. Deparaffinized sections were labeled with the FGFR1/KLB or VE‐cadherin/KLB antibodies. The cell nuclei were labeled with DAPI. For the negative controls, blocking solution was utilized to replace the primary antibody.

### Western blot analysis

After the indicated treatment, the cells were washed with PBS, and the protein extracts were harvested by adding a RIPA lysis buffer system (containing lysis buffer, PMSF, protease inhibitor cocktail, and sodium orthovanadate; Santa Cruz Biotechnology, SC‐24948). The detailed procedure followed a general protocol described elsewhere 4.

### Immunofluorescence staining

Immunofluorescence staining of HMVECs cultured on eight‐well culture slides (Corning, Discovery Labware, Oak Park, MA, USA) and mouse heart sections followed a method described in previous studies 4.

### Duolink *in situ* assay

A Duolink *in situ* proximity ligation assay (PLA) was performed following the manufacturer's instructions (Sigma‐Aldrich). Briefly, HMVECs were treated with N‐FGFR1 (1.5 μg·mL^−1^) or TGFβ2 (5 ng·mL^−1^) for 48 h with or without a 2‐h preincubation with AcSDKP (100 nm). Normal isotype IgG was used as the control. After washing with PBS, the cells were fixed with 4% paraformaldehyde and permeabilized with 0.2% Triton X‐100. After blocking, the cells were incubated overnight with primary antibodies against FGFR1 and KLB. Cells without the addition of a primary antibody were used as the negative control. The cells were incubated with the PLA probe solution for 1 h at 37 °C, followed by incubation with a ligase solution for 30 min at 37 °C and a polymerase amplification solution for 100 min at 37 °C. The samples were then mounted with Duolink *in situ* mounting medium with DAPI for 20 min and analyzed by using an all‐in‐one BZ‐X700 fluorescence microscope (Keyence, Osaka, Japan). For each slide, the images in at least six different fields at ×400 magnification were analyzed 4.

### Immunoprecipitation

Human dermal microvascular endothelial cells were subjected to the indicated treatment for 48 h and harvested by the RIPA lysis buffer system and centrifugation; the immunoprecipitation (IP) assay was then performed according to the manufacturer's instructions (Cell Signaling Technology). The detailed procedure was as described in previous studies 4.

### Animal experiments

Control, streptozotocin (STZ)‐treated CD‐1, and AcSDKP‐treated STZ mice were prepared according to our previous study 9, and the characteristics of animals were as reported 4. In brief, 8‐week‐old male CD‐1 mice were subjected to a single intraperitoneal injection of STZ (200 mg·kg^−1^) to induce diabetes. Twelve weeks later, the diabetic mice were divided into a nontreatment group and an AcSDKP treatment group (500 μg·kg BW^−1^·day^−1^ using an osmotic mini‐pump) for another 4 weeks. The experiments described in the methods were carried out in compliance with the animal protocols of Kanazawa Medical University (protocol numbers 2014‐89, 2013‐114, and 2014‐101).

### Statistical analysis

The graphs were generated, and statistical analyses were performed using graphpad prism software (Version 7.00, La Jolla, Canada). The data are presented as the means ± SDs. One‐way ANOVA followed by Tukey's multiple comparisons test was used for statistical analysis. A *P*‐value of < 0.05 was considered significant. All data were confirmed in three independent experiments.

## Results

### AcSDKP induced the FGFR1–KLB complex in endothelial cells

**Figure 1:**
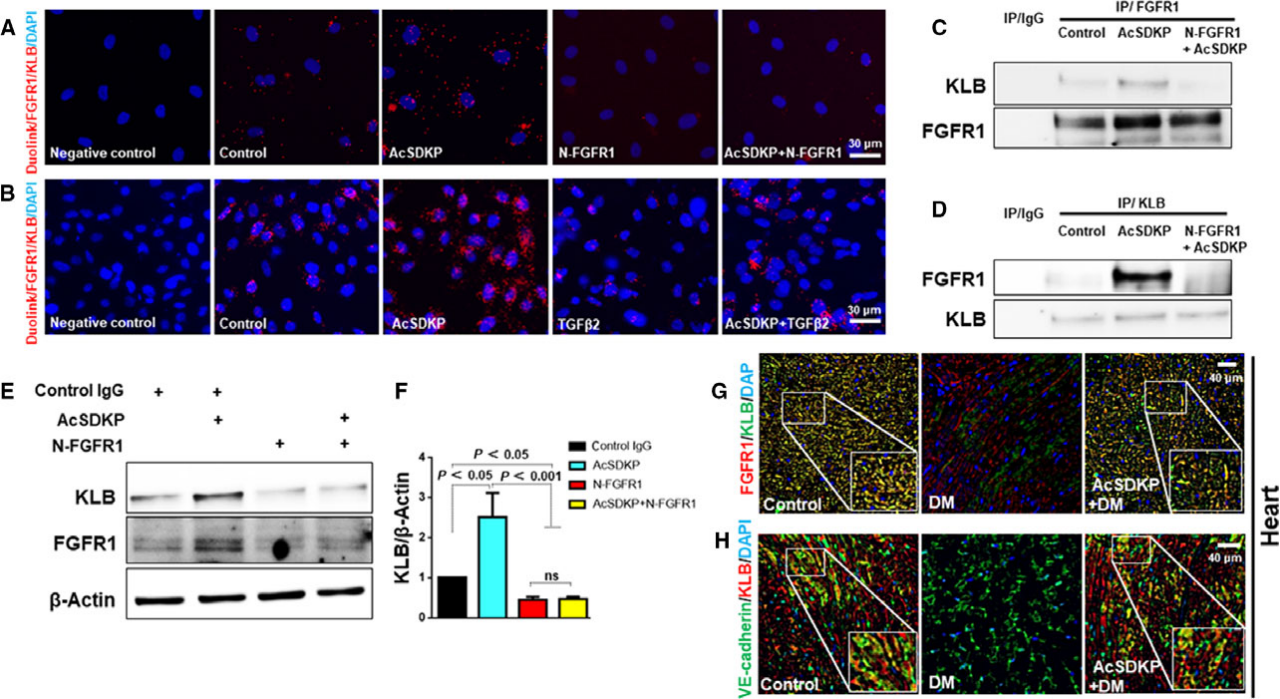
AcSDKP increased the FGFR1–KLB complex in endothelial cells. HMVECs were subjected to treatment with N‐FGFR1 (1.5 mg·mL^−1^) or TGFβ2 (5 ng·mL^−1^) for 48 h either with or without preincubation with AcSDKP (100 nm) for 2 h. (A, B) A Duolink *in situ* PLA was used to determine the proximity between FGFR1 and KLB (×200; scale bar, 30 μm). Negative control: cells without primary antibodies against FGFR1 and KLB in the PLA assay. Six different fields in each slide were evaluated. The cell lysates were subjected to IP with normal IgG or anti‐FGFR1 (C)/anti‐KLB (D) antibodies, followed by western blot analysis with antibodies against KLB or FGFR1. (E) Western blot analysis of the FGFR1 and KLB protein levels in cells treated with N‐FGFR1 (1.5 mg·mL^−1^) for 48 h with or without preincubation with AcSDKP (100 nm) for 2 h; KLB levels are quantified in (F). Data are mean ± SD. *n* = 3 in each group were carried out. The statistical significance was evaluated by using one‐way ANOVA followed by Tukey's multiple comparisons. Immunofluorescence staining of FGFR1/KLB (G) and VE‐cadherin/KLB (H) in mouse heart sections was carried out (×200; scale bar, 40 μm). For each slide, images of six different fields were evaluated. Diabetic mice were abbreviated as DM in the figures.

We have previously demonstrated that the anti‐EndMT effects of AcSDKP mediated by the suppression of the TGFβ/Smad pathway were associated with the induction of FGFR1 signaling 4, 9. However, it remains unclear whether KLB, the cofactor of FGFRs, is involved in this process. First, we performed the proximity assay and found that endogenous FGFR1 and KLB are expressed physically near each other in cultured endothelial cells (Fig. 1A,B). In addition, AcSDKP treatment significantly enhanced the proximity of FGFR1 and KLB. The proximity of FGFR1 and KLB was abolished by the co‐incubation with neutralizing FGFR1 antibody (N‐FGFR1; Fig. 1A) or TGFβ2 (Fig. 1B). The decreased proximity of FGFR1 and KLB induced by N‐FGFR1 was not altered by AcSDKP; TGFβ‐induced disruption of the proximity of FGFR1 and KLB was partially rescued by preincubation with AcSDKP (Fig. 1A,B).

A direct physical interaction between KLB and FGFRs was confirmed by Ogawa *et al*. 33. Consistent with Ogawa's report, our IP results revealed a physical interaction between KLB and FGFR1 in normal endothelial cells. IP analysis demonstrated that AcSDKP induced the FGFR1–KLB complex; co‐incubation with N‐FGFR1 abolished AcSDKP‐enhanced levels of FGFR1–KLB complex in AcSDKP‐treated endothelial cells (Fig. 1C). A comparable result was obtained when the anti‐KLB antibody was used as the pull‐down antibody (Fig. 1D). The levels of KLB and FGFR1 were elevated upon AcSDKP treatment; AcSDKP did not affect the levels of either FGFR1 or KLB in N‐FGFR1‐treated cells (Fig. 1E,F), suggesting that AcSDKP increased KLB in an FGFR1‐dependent manner and subsequent induction of FGFR1–KLB complex.

We have previously reported that FGFR1 expression was reduced in the heart of STZ‐induced diabetes model animals and that the administration of AcSDKP could restore FGFR1 expression 4, 9. As expected, a notable decrease in the FGFR1 and KLB levels was observed in the hearts of STZ‐induced diabetic mice compared to the levels of these proteins in the hearts of control mice; administration of AcSDKP significantly restored them (Fig. 1G). In line with this finding, VE‐cadherin‐positive endothelial cells in the hearts of diabetic mice exhibited reduced levels of KLB; however, AcSDKP restored the KLB levels (Fig. 1H).

### KLB deficiency led to EndMT in HMVECs

**Figure 2:**
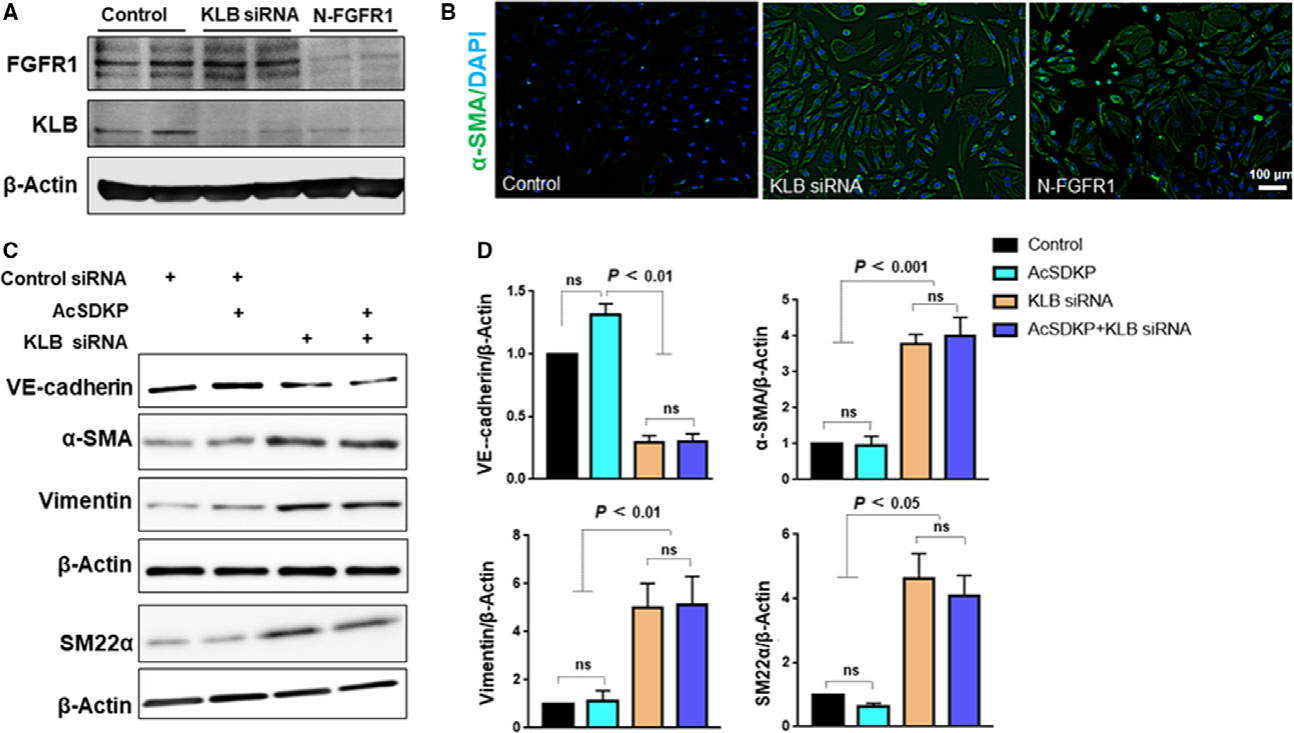
KLB deficiency led to EndMT in HMVECs. (A) Subconfluent HMVECs were transfected with KLB siRNA or control siRNA. Six hours later, the medium was replaced with an experimental medium, followed by N‐FGFR1 (1.5 mg·mL^−1^) treatment. At 48 h, the cells were harvested for western blot analysis. The results are from three repeated experiments. (B) The same treated HMVECs (as in A) cultured on 8‐well culture slides were subjected to immunofluorescence staining with an anti‐α‐SMA antibody and DAPI (scale bar, 100 μm). Six different fields were observed for each slide. (C) HMVECs with or without preincubation with AcSDKP (100 nm) for 2 h were transfected with KLB siRNA or control siRNA for 48 h. The expression of EndMT markers, including VE‐cadherin, α‐SMA, vimentin, and SM22α, was assessed by western blotting and quantified (D) by imagej software (GE Healthcare Life Sciences, Uppsala, Sweden). The data represent mean ± SD and are representative of three independent experiments. One‐way ANOVA with Tukey's multiple comparisons test was used for statistical analysis.

First, we confirmed the levels of KLB and FGFR1 in cells treated either with specific siRNA targeting KLB or with N‐FGFR1. N‐FGFR1 diminished the levels of both FGFR1 and KLB; KLB siRNA‐transfected cells exhibited decreased levels of KLB but no alteration in FGFR1 levels (Fig. 2A). Immunofluorescence analysis revealed that a deficiency in either KLB or FGFR1 induced an increase in αSMA levels and an alteration of the cell morphology to a spindle shape (Fig. 2B). Consistent with this observation, we found that specific siRNA‐mediated KLB knockdown resulted in the induction of EndMT, as evidenced by the decreased VE‐cadherin levels and enhanced expression of αSMA, SM22α, vimentin, and FSP1; however, AcSDKP had no effect on KLB deficiency‐induced EndMT (Fig. 2C,D).

### KLB deficiency was not associated with the induction of TGFβ/Smad3 signaling

**Figure 3:**
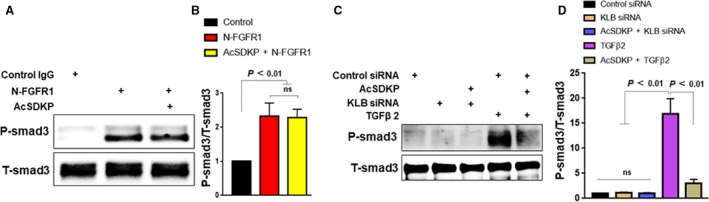
KLB deficiency was not associated with the induction of TGFβ/Smad3 signaling HMVECs were treated with control IgG or N‐FGFR1 for 48 h with or without preincubation with AcSDKP (100 nm) for 2 h; the phosphorylation of Smad3 (P‐Smad3) was evaluated by western blot analysis (A), and P‐Smad3 expression was normalized to total Smad3 (T‐Smad3) expression by imagej software (B). HMVECs were subjected to KLB siRNA or TGFβ2 (5 ng·mL^−1^) treatment for 48 h with or without preincubation with AcSDKP (100 nm) for 2 h; the activation of P‐Smad3/T‐Smad3 was examined by western blot analysis (C) and quantified (D). The data represent mean ± SD (*n* = 3). The statistical analysis was carried out by utilizing one‐way ANOVA with Tukey's multiple comparisons test.

Fibroblast growth factor receptor 1 suppression‐induced EndMT has shown to be associated with the activation of the TGFβ/Smad3 pathway 4, 5, 9. As reported elsewhere, cells incubated with N‐FGFR1 exhibited increased phosphorylation of Smad3 compared to that in cells incubated with control IgG (Fig. 3A,B); as reported, AcSDKP did not affect Smad3 phosphorylation in N‐FGFR1‐treated cells (Fig. 3A,B). TGFβ2‐treated cells were used as the positive control for the phosphorylation of Smad3, and AcSDKP suppressed TGFβ2‐induced Smad3 phosphorylation (Fig. 3C,D) 4. However, unexpectedly, KLB knockdown did not impact on the Smad3 phosphorylation in HMVECs (Fig. 3C,D).

### KLB deficiency‐induced EndMT is dependent on the mitogen‐activated protein kinase pathway

**Figure 4:**
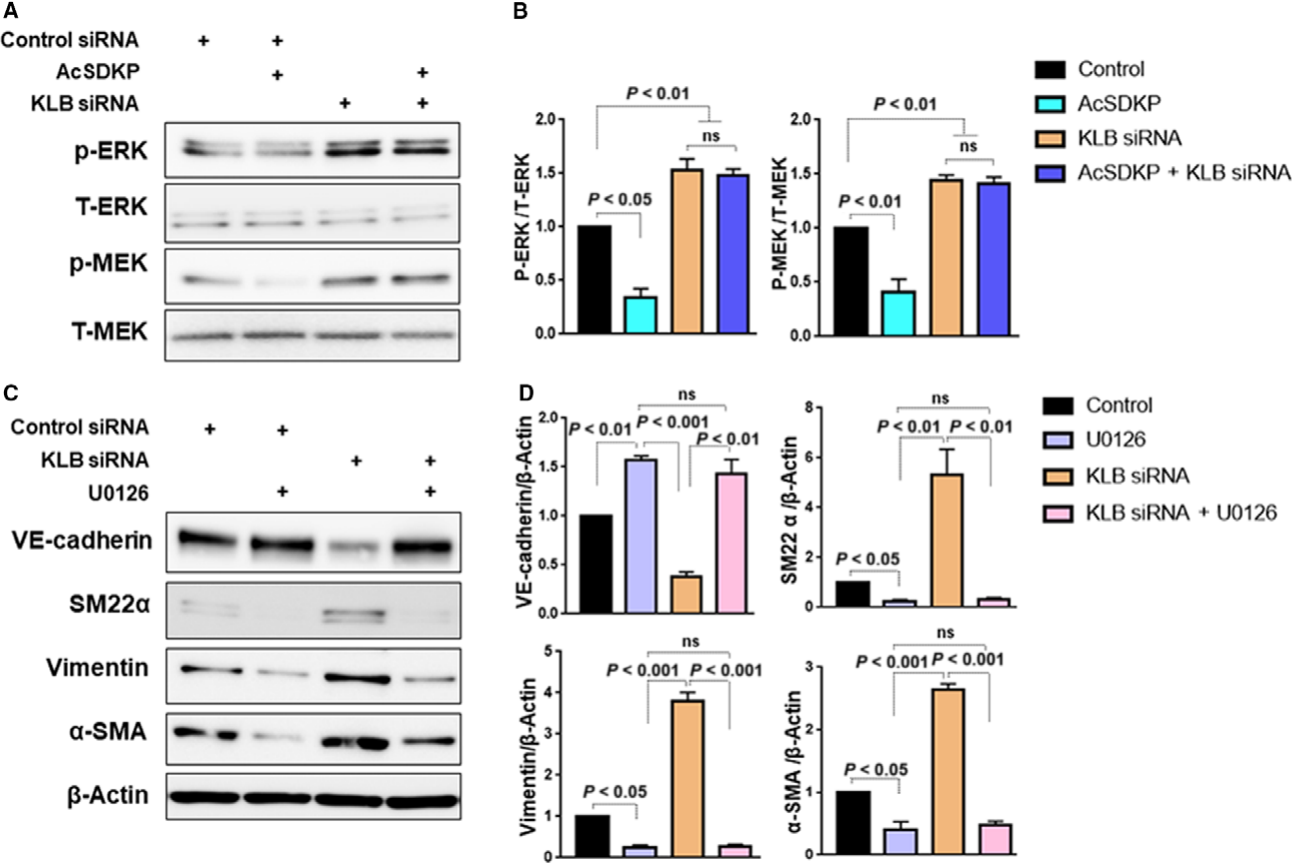
KLB deficiency‐induced EndMT is dependent on the MAPK pathway. HMVECs were transfected with KLB siRNA (or control siRNA) for 48 h with or without preincubation with AcSDKP (100 nm) for 2 h; the levels of phosphorylated ERK and MEK (P‐ERK and P‐MEK) and the levels of total ERK and MEK (T‐ERK and T‐MEK) were examined by western blot analysis (A) and quantified (B). The expression of EndMT markers in cells transfected with KLB siRNA in the presence or absence of U0126 (10 μm, MEK inhibitor) was determined by western blotting (C) and quantified (D). The data represent mean ± SD. Three independent experiments were performed for each result. ANOVA with Tukey's multiple comparisons test was applied.

Liu *et al*. 34 recently reported that the overexpression of KLB in endometrial adenocarcinoma cells inhibited 17β‐estradiol‐induced epithelial–mesenchymal transition (EMT) via the suppression of ERK1/2. In basal condition, AcSDKP suppressed both MEK and ERK phosphorylation in culture endothelial cells (Fig. 4A,B). KLB knockdown by specific siRNA induced a significant increase in the phosphorylation of MEK and ERK; AcSDKP did not inhibit KLB deficiency‐induced activation of the MEK/ERK pathway (Fig. 4A,B). Consistent with this finding, the MEK inhibitor U0126 completely inhibited KLB deficiency‐induced EndMT (Fig. 4C,D), suggesting the fundamental role of the MEK/ERK pathway in KLB deficiency‐induced EndMT. Supporting with this, the occurrence of EndMT and the activation of MEK/ERK pathway induced by N‐FGFR1 were suppressed by U0126. (Fig. S1A–D).

### Endocrine FGF enhanced anti‐EndMT effect of AcSDKP

**Figure 5:**
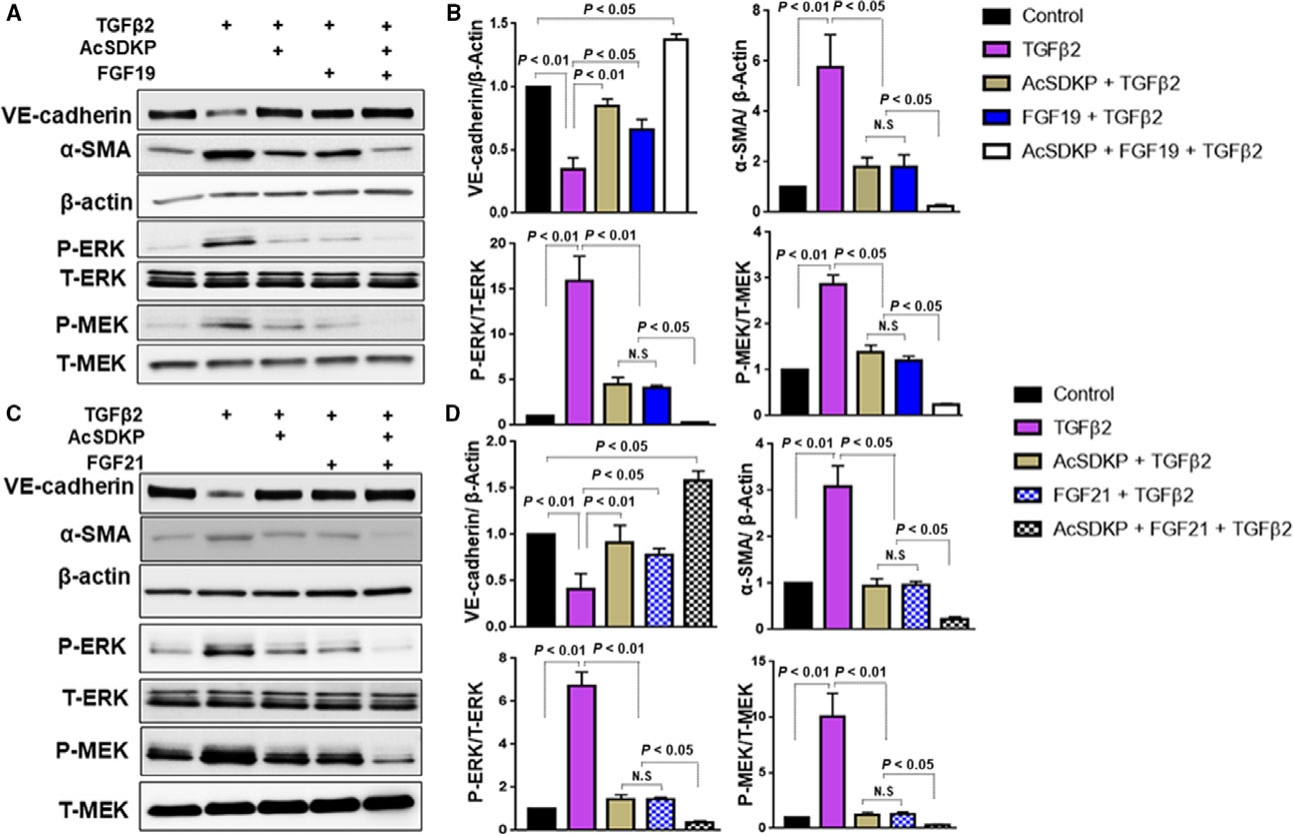
Either FGF19 or FGF21 enhanced the inhibitory effect of AcSDKP on EndMT and MEK/ERK pathway. In the presence of AcSDKP, HMVECs were stimulated by TGFβ2 with or without FGF19 (100 ng·mL^−1^) or FGF21 (100 ng·mL^−1^) treatment. The levels of VE‐cadherin/β‐Actin, α‐SMA/β‐Actin, P‐ERK/T‐ERK, and P‐MEK/T‐MEK were examined by western blot analysis (A, C) and quantified (B, D). The data represent mean ± SD. Three independent experiments were performed for each result. ANOVA with Tukey's multiple comparisons test was applied.

Both endocrine FGF19 and FGF21 work on FGFRs (FGFR1c, 2c and 3c) with tyrosine kinase activity through binding to cell surface proteins KLB 35, 36, 37, 38, 39, 40, 41. We finally evaluated whether FGF19 or FGF21, the ligands of FGFR1/KLB, displayed anti‐EndMT effects via suppression of MEK/ERK pathway. The incubation with either FGF19 or FGF21 inhibited TGFβ2‐induced EndMT as similar to levels in the cell treated with AcSDKP (Fig. 5A–D). FGF19 or FGF21 enhanced the inhibitory effects of AcSDKP on TGFβ2‐induced EndMT and MEK/ERK pathway (Fig. 5A–D).

## Discussion

Combating organ fibrosis is an important topic in medical research. The attractive anti‐EndMT effects of AcSDKP have been intensively reported by our group and other groups during the last decade 4, 9, 10, 11, 12. The induction of FGFR1 expression upon AcSDKP treatment has been considered the critical event responsible for the inhibition of the TGFβ/Smad signaling pathway and fibrosis 4, 9, 11. In current study, we demonstrated that (a) AcSDKP increased the level of KLB via an FGFR1‐dependent manner and subsequent induction of FGFR1–KLB complex in endothelial cells; (b) KLB deficiency did not influence FGFR1 levels in endothelial cells; (c) KLB deficiency‐induced Smad3 signaling‐independent but MAPK‐dependent EndMT; (d) AcSDKP had no effect on KLB deficiency‐induced EndMT; and (d) either FGF19 or FGF21 enhanced anti‐EndMT effect of AcSDKP. These data provided deeper understanding of the molecular mechanisms of the powerful endogenous antifibrotic molecule AcSDKP.

Neither AcSDKP‐mediated‐regulation of KLB nor ‐influence on the level of FGFR1–KLB complex has been studied. In our study, AcSDKP increased the levels of KLB and subsequent induction of FGFR1–KLB complex; AcSDKP had no effect on the decreased levels of KLB in N‐FGFR1‐treated endothelial cells, suggesting that AcSDKP‐induced KLB expression is FGFR1‐dependent. The reduction in the FGFR1 and KLB protein levels was also observed in the hearts of STZ‐induced diabetic mice, and AcSDKP restored these levels. A logical question is whether KLB affects the FGFR1 level in endothelial cells; however, we found that the suppression of KLB by specific siRNA did not influence the FGFR1 levels indicating that the FGFR1 level is not regulated by the KLB protein in cultured endothelial cells.

We next explored the signaling pathway through which EndMT is induced by KLB knockdown. Considering the activation of TGFβ/Smad3 signaling upon FGFR1 deficiency, the phosphorylation of Smad3 was first analyzed in KLB knockdown endothelial cells. Surprisingly, no obvious alteration in Smad3 phosphorylation was observed. Instead, molecules in the MAPK signaling pathway, MEK1/2 and ERK1/2, were activated, and the MEK inhibitor significantly blocked KLB suppression‐induced EndMT. This observation was consistent with Liu's report, which showed that the overexpression of KLB abolished the activation of ERK1/2 and EMT, the epithelial program sharing similar molecular mechanisms to EndMT 34. Furthermore, our data demonstrated that the activation of the MAPK pathway without the activation of the Smad3 signaling pathway in KLB‐deficient cells is sufficient to induce EndMT. Regard with this, ligand of FGFR1–KLB complex, either FGF19 or FGF21, could synergistically suppress EndMT and MEK/ERK pathway in AcSDKP‐treated cells, suggesting the biological significance of AcSDKP in endothelial cells through the induction of FGFR1–KLB complex.

In conclusion, the current study highlights the involvement of KLB in EndMT pathobiology and in the anti‐EndMT effects of AcSDKP. These data provide new insight into the essential role of KLB, which is important for establishing new therapeutic strategies to combat progressive fibrotic diseases associated with EndMT.

## Conflict of interest

The authors declare no conflict of interest.

## Author contributions

RG performed all the experiments and contributed to manuscript writing. KK proposed the original idea, designed and supervised the experiments, provided intellectual input, and edited the manuscript. JL participated in the establishment of the diabetic mouse model and immunofluorescence staining. MK and TO were involved in the discussions. DK provided intellectual input.

## Supporting information


**Fig S1**. MEK inhibitor U0126 blunted the N‐FGFR1‐induced EndMT and activation of MEK/ERK pathway. HMVECs were treated with control IgG or N‐FGFR1 for 48 h with or without U0126 treatment. The VE‐cadherin/β‐Actin, SM22α/β‐Actin, P‐ERK/T‐ERK and P‐MEK/T‐MEK were evaluated by western bolt analysis and quantified (A–D). The data represent mean ± SD (*n* = 3). The statistical analysis was carried out by utilizing one‐way ANOVA with Tukey's multiple comparisons test.
