# Supplementary figures and images for: βklotho is essential for the anti‐endothelial mesenchymal transition effects of N‐acetyl‐seryl‐aspartyl‐lysyl‐proline
Source: FEBS Open Bio. 2019 Apr 22;9(5):1029–38. doi: 10.1002/2211-5463.12638 (PMC6487725; doi:10.1002/2211-5463.12638)

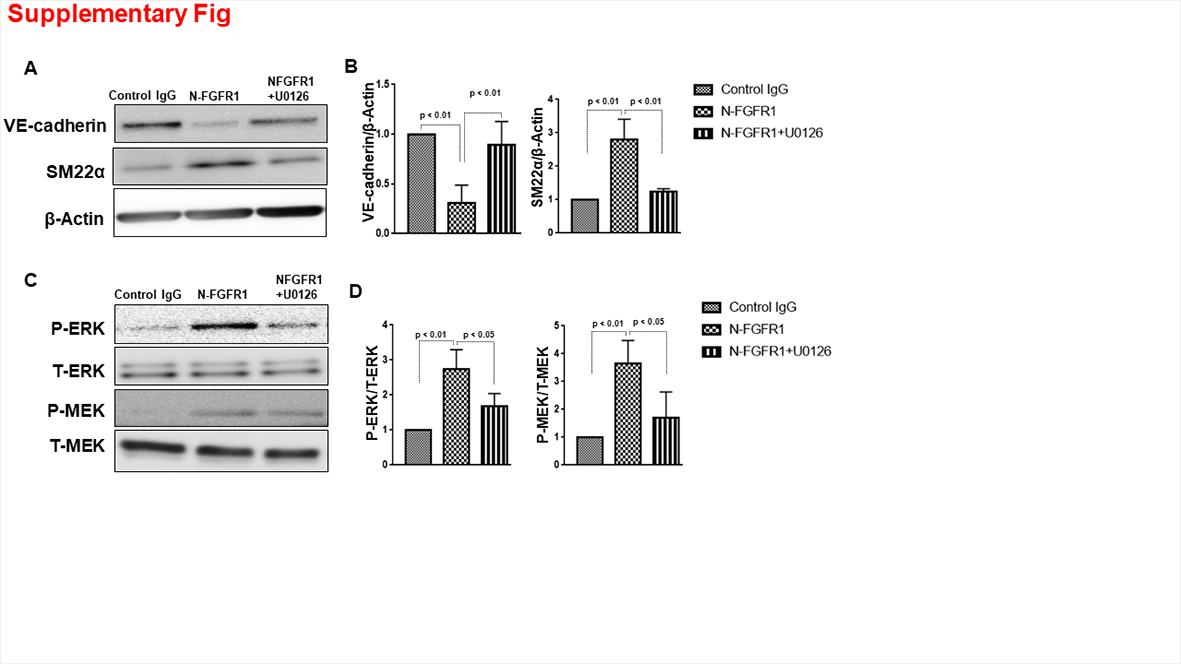

Supplement: Supplementary file 1 — Fig S1. MEK inhibitor U0126 blunted the N‐FGFR1‐induced EndMT and activation of MEK/ERK pathway. HMVECs were treated with control IgG or N‐FGFR1 for 48 h with or without U0126 treatment. The VE‐cadherin/β‐Actin, SM22α/β‐Actin, P‐ERK/T‐ERK and P‐MEK/T‐MEK were evaluated by western bolt analysis and quantified (A–D). The data represent mean ± SD (n = 3). The statistical analysis was carried out by utilizing one‐way ANOVA with Tukey's multiple comparisons test. [file FEB4-9-1029-s001.tif]
